# Novel laparoscopic renal denervation immediately reduces atrial fibrillation inducibility: a swine model study

**DOI:** 10.1038/s41598-023-47077-w

**Published:** 2023-11-11

**Authors:** Soonil Kwon, Eue-Keun Choi, Hyo-Jeong Ahn, So-Ryoung Lee, Seil Oh, Si Hyun Kim, Minh-Tung Do, Jang Hee Han, Chang Wook Jeong

**Affiliations:** 1 Division of Cardiology, Department of Internal Medicine, Seoul National University Hospital, Seoul, Republic of Korea; 2 Department of Internal Medicine, Seoul National University College of Medicine, 101 Daehak-ro, Jongno-gu, Seoul, 03080 Republic of Korea; 3 Department of Urology, Seoul National University Hospital, Seoul, Republic of Korea; 4 Department of Surgery, Hai Phong University of Medicine and Pharmacy, Hai Phong, Vietnam; 5 Department of Urology, Seoul National University College of Medicine, 101 Daehak-ro, Jongno-gu, Seoul, 03080 Republic of Korea

**Keywords:** Interventional cardiology, Atrial fibrillation

## Abstract

Catheter-based approaches may have inherent limitations in achieving effective renal denervation (RDN) and treatment of atrial fibrillation (AF). This study aimed to investigate the acute effects of novel laparoscopic RDN on modulating AF inducibility using a swine model. Four and five swine were randomly allocated to the sham and RDN groups, respectively. Each swine underwent measurement of the atrial effective refractory period (AERP) and AF induction tests using burst atrial pacing before and immediately after sham or RDN procedures with and without vagal nerve stimulation (VNS). A laparoscopic RDN procedure circumferentially ablated the renal nerves round the renal arteries using radiofrequency energy. There was no significant difference in the baseline AERP between the two groups (p > 0.05). Under VNS, AERP was significantly increased by 20 ms after laparoscopic RDN (95% CI = 0–30, p = 0.004). Compared to the sham group, the RDN group showed significantly reduced AF inducibility [OR (95% CI) = 0.32 (0.13–0.76) and 0.24 (0.11–0.57) with and without VNS, respectively]. After laparoscopic RDN, the duration of inducible AF episodes was significantly shortened from 28 (10–77) s to 7 (3–11) s (p < 0.001). The novel laparoscopic RDN can immediately reduce AF inducibility in a swine model.

## Introduction

Atrial fibrillation (AF) is the most common arrhythmia, and its prevalence among the elderly is expected to increase by 1.8-fold by 2060^1^. Although recent advances in catheter ablation have improved the management of patients with AF^2^, recurrence is of concern due to pulmonary vein reconnection and atrial substrate progression^3^. Various strategies for catheter ablation have been suggested, but consensus is lacking on the optimal strategy^2^. Therefore, adjunctive therapy is required to improve rhythm management in AF.

Renal denervation (RDN) has been introduced to treat uncontrolled hypertension by removing sympathetic modulation mediated by the renal sympathetic nerves. Several studies have shown the effect of RDN in treating hypertension^4–6^. RDN has also been studied as an adjunctive therapy to catheter ablation for AF. It showed a promising result in decreasing the recurrence risk by adding to conventional pulmonary vein isolation^7^. However, contradictory findings exist regarding the therapeutic effect of RDN^8–10^, which may suggest that a current RDN procedure requires improvement.

Most current commercial RDN systems use a catheter-based approach to deliver radiofrequency or ultrasound energy from inside the renal artery to ablate the renal sympathetic nerves. However, the usefulness of this approach is limited in unfavorable conditions such as renal artery stenosis, significant atherosclerosis, vessel calcification, or anomalous anatomy. In addition, the effectiveness of a catheter-based RDN may be limited because 16% of nerve fibers are located ≥ 3 mm away from the endoluminal surface^11^. Thus, the catheter-based approach may have an inherent limitation in effectively ablating renal sympathetic nerves because they often present as thick bundles and form a web-like network to course spirally along the renal artery. To overcome these limitations, we recently developed a novel laparoscopic RDN system that is minimally invasive and effective for ablating renal sympathetic nerves^12,13^.

This study aimed to validate the acute impact of a novel laparoscopic RDN procedure on the modulation of AF inducibility in a swine model.

## Results

Nine male swine with a median weight of 38 kg (interquartile range, 38–42 kg) were used in the experiment. Among the swine, four and five were assigned to the sham and RDN groups, respectively. Both procedures were successful without acute periprocedural complications. The median procedure times for sham and laparoscopic RDN procedures were 30 and 66 min, respectively. The time between the procedure and the post-procedural electrophysiology study was a median of 60 min (30–60 min). Cervical-level vagal nerve stimulation (VNS) was successful in all swine without acute periprocedural complications. Compared to the baseline, VNS resulted in a median decrease of -10 per minute in heart rate: 89 (85–95) per minute versus 98 (96–111) per minute, respectively (p < 0.001).

### Prolonged AERP after laparoscopic RDN

**Figure 1:**
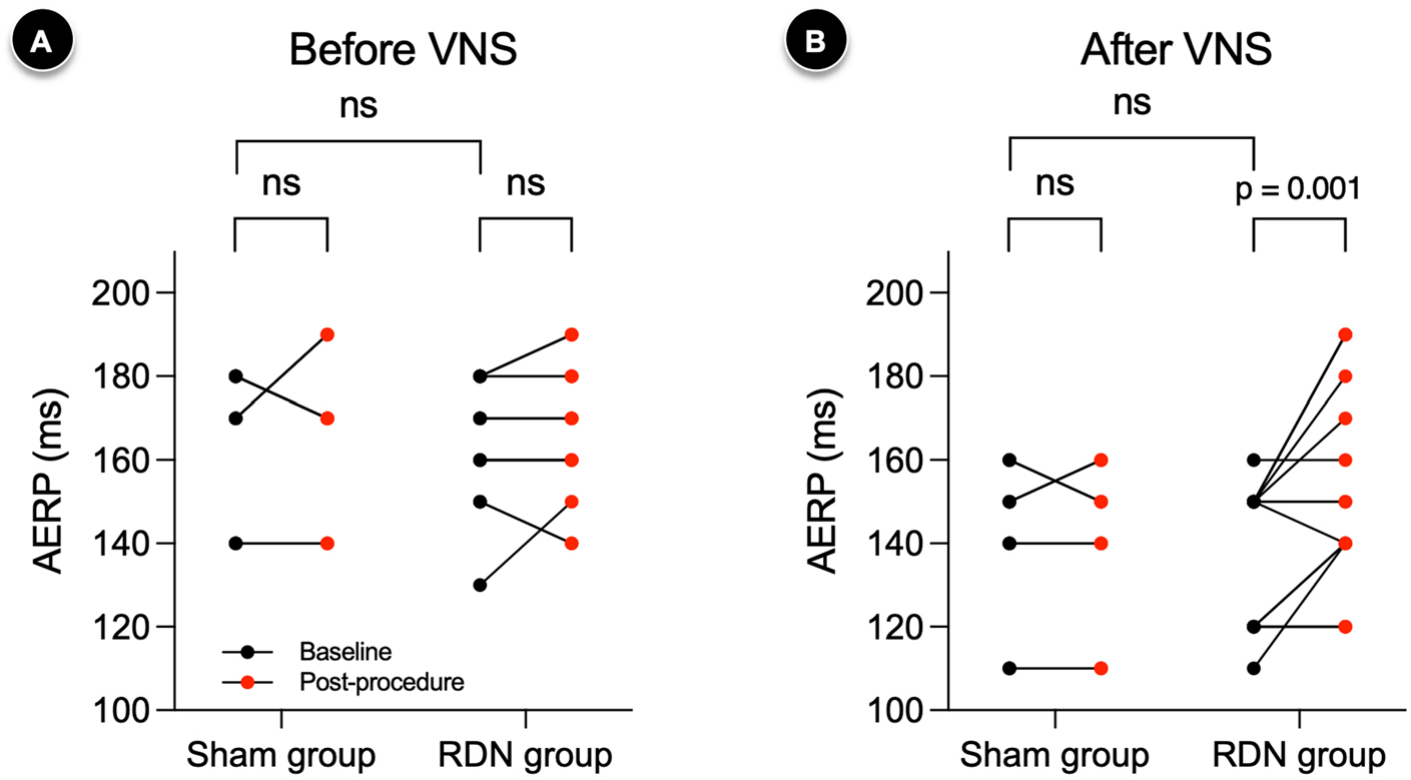
Changes in AERPs according to procedures and VNS. Compared to the sham procedure, laparoscopic RDN significantly increased AERP after VNS. The number of data points in each group may not match the number of animals used (n = 4 for the sham group, n = 5 for the RDN group) due to the overlap of data points. The Wilcoxon signed-rank test was performed to compare the pair of AERPs obtained before and after the sham or laparoscopic RDN procedure. ns : p-value ≥ 0.05. *AERP* atrial effective refractory period, *RDN* renal denervation, *VNS* vagal nerve stimulation.

The baseline atrial effective refractory periods (AERPs) before VNS were 170 (140–180) ms and 170 (160–180) ms in the sham and RDN groups, respectively, which were comparable (p = 0.726). VNS showed a general effect of decreasing AERP, regardless of the procedure and group. VNS significantly reduced the baseline AERP by 30 (95% CI, 10–40) ms in both groups (both p < 0.001). A similar trend was also observed in the post-procedural AERP, with a decrease of 30 ms (95% CI, 10–40; p < 0.001) and 10 ms (95% CI, 0–20; p = 0.004) for the sham and RDN groups, respectively. Before VNS, the sham (p = 0.824) and RDN groups (p = 0.531) did not show a significant difference in AERP between the baseline and post-procedural measurements (Fig. 1A). However, after VNS, AERP was significantly increased only after laparoscopic RDN by 20 ms (95% CI, 0–30; p = 0.001) (Fig. 1B). In contrast, no significant difference was noted in the AERP after the sham procedure (p > 0.999).

### Reduced AF inducibility after laparoscopic RDN

AF induction tests were repeated ten times in each session, and AF episodes longer than 5 s were counted to calculate AF inducibility. For the sham group, the sham procedure did not significantly change AF inducibility regardless of VNS, with OR 1.35 (95% CI, 0.55–3.16; p = 0.655) and 1.25 (95% CI, 0.52–3.09; p = 0.815) before and after VNS, respectively. However, laparoscopic RDN significantly changed AF inducibility by -24.0% and -34.0% before and after VNS with an OR of 0.32 (95% CI, 0.13–0.76; p = 0.018) and 0.24 (0.11–0.57; p = 0.001), respectively.

**Figure 2:**
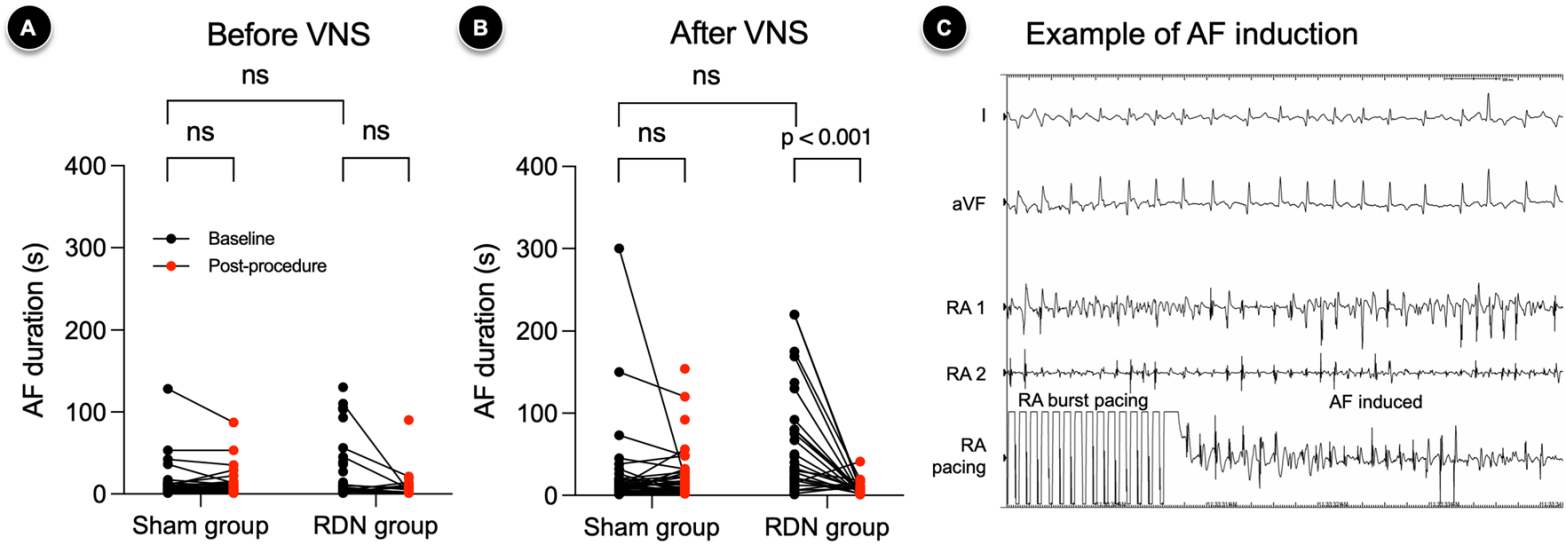
Durations of inducible AF episodes according to procedures and VNS. Compared to the sham procedure, laparoscopic RDN significantly reduced durations of inducible AF episodes after VNS. (**A,B**) Inducible AF episodes before and after VNS. (**C**) An example of AF induction by RA burst pacing. The number of data points in each group may not match the number of animals used (n = 4 for the sham group, n = 5 for the RDN group) due to the overlap of data points. The Wilcoxon signed-rank test was performed to compare the pair of AERPs obtained before and after the sham or laparoscopic RDN procedure. ns : p-value ≥ 0.05. *AF* atrial fibrillation, *RA* right atrial, *RDN* renal denervation, *VNS* vagal nerve stimulation.

Before VNS, the sham and laparoscopic RDN procedures did not significantly change the AF durations, although they were marginally decreased after laparoscopic RDN from 11 s (5–98) to 5 s (3–10) (p = 0.076) (Fig. 2A). The effect of laparoscopic RDN on decreasing AF duration accentuated after VNS. After VNS, laparoscopic RDN significantly decreased AF duration from 28 s (10–77) to 7 s (3–11) (p < 0.001) (Fig. 2B). In contrast, there was no significant difference in the AF duration after the sham procedure (p = 0.706). An example of AF induction by right atrial burst pacing is presented in Fig. 2C.

### Effectively ablated renal sympathetic nerves by laparoscopic RDN

**Figure 3:**
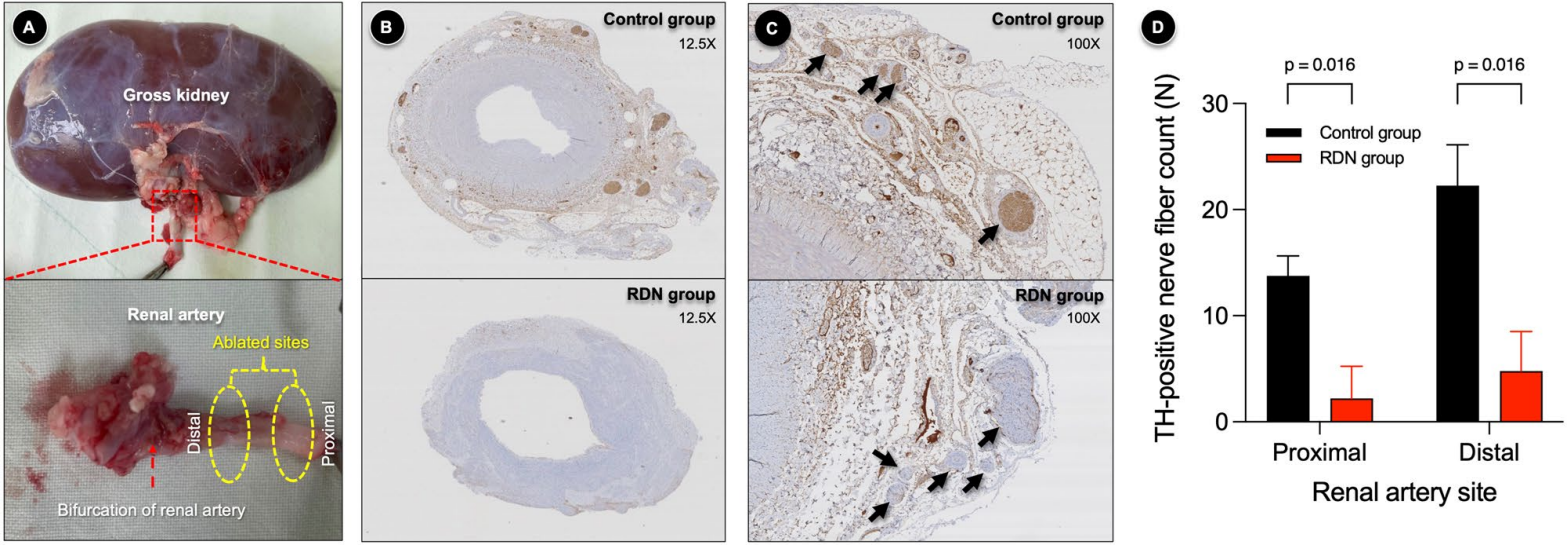
Renal sympathetic nerves ablated by laparoscopic RDN. (**A**) Laparoscopic RDN ablated renal sympathetic nerves at the proximal and distal sites of the renal artery. (**B**) Cross-sectional slides of the renal artery after dissecting periarterial tissues. Despite laparoscopic RDN, there was no significant vessel wall deformity or injury of tunica media. (**C**) A comparison of the adventitia of the renal artery between the sham and RDN groups. Immunohistochemistry of TH was performed to identify functional renal sympathetic nerves. TH-positive renal nerves were destructed in the RDN group whereas they were intact in the sham group (arrows). (**D**) Compared to the sham group, the RDN group was associated with a significantly reduced number of TH-positive renal nerve fibers, for both proximally and distally ablated sites. The Mann–Whitney *U*-test was performed to compare nerve fiber counts between the sham (4 pairs of right and left renal arteries obtained from 4 swine) and RDN (5 pairs of right and left renal arteries obtained from 5 swine) groups. *RDN* renal denervation, *TH* tyrosine hydroxylase.

We harvested both the right and left kidneys and obtained renal arteries from all swine. On gross inspection, the periarterial subcutaneous tissues were well dissected along the ablated sites (Fig. 3A). Representative cross-sectional slides of proximal and distal sites of the renal artery are shown in Supplemental Fig. 1. Cross-sectional slides of the renal artery showed no significant vessel deformity or tunica media injury due to laparoscopic RDN (Fig. 3B). Immunohistochemical analysis of tyrosine hydroxylase (TH) to identify functional renal sympathetic nerves showed that TH-positive renal nerves were destroyed in the RDN group but were intact in the sham group (Fig. 3C). TH-positive nerve fibers were counted along the renal artery adventitia in both the groups. The number of TH-positive nerve fibers was significantly lower in the RDN group than in the sham group (median 0 versus 15 and 5 versus 21 at the proximal and distal sites, respectively; p = 0.016) (Fig. 3D).

## Discussion

This study investigated the acute impact of a novel laparoscopic RDN on AF inducibility using a swine model. The main findings are summarized as follows: (1) Compared to the sham procedure, laparoscopic RDN significantly prolonged AERP under VNS; (2) laparoscopic RDN significantly reduced AF inducibility regardless of VNS; (3) laparoscopic RDN significantly decreased inducible AF durations; and (4) laparoscopic RDN procedure effectively ablated renal sympathetic nerves without damage to the renal artery, and it showed an acute effect on modulating AF inducibility.

In our study, AERP was significantly prolonged by 30 ms after laparoscopic RDN under VNS only. In contrast, there was no significant difference in AERP between the sham procedure and laparoscopic RDN without VNS. This finding suggests that the laparoscopic RDN procedure may mitigate the effect of the vagus nerve activation that decreases AERP, which risks AF inducibility^3^. Shortened AERP is related to electrical remodeling induced by AF but also it increases the susceptibility of inducible AF^14^. Therefore, laparoscopic RDN may reduce AF risks by increasing AERP, especially among patients with vagally mediated AF^15^, which may account for up to 27% of paroxysmal AF^16^. High-intensity vagal tone decreases AERP. At the same time, it increases the dispersion of AERP, causing an electrically favorable environment to AF^17^. Therefore, a blockade of increased vagal tone has a potential therapeutic effect in preventing AF^18^. Without VNS, the AERP might not be affected immediately after laparoscopic RDN in our study because reverse atrial remodeling requires some periods, even if RDN had successfully modulated autonomic modulation of the heart^19^. Therefore, we expect that laparoscopic RDN would also increase AERP without VNS in the long term, and further study is required to verify the long-term effect of laparoscopic RDN. Unlike AERP, AF inducibility acutely decreased after laparoscopic RDN, regardless of VNS. Burst atrial pacing increases the heterogeneity of atrial sympathetic innervation and promotes AF^20^. Therefore, laparoscopic RDN may reduce AF inducibility regardless of VNS by altering the effect of burst atrial pacing.

Although a catheter-based RDN has been suggested as an adjunctive therapeutic modality for AF^21^, there have been contradictory results^10^. A catheter-based approach has been introduced as an alternative to a surgical approach because surgical removal of thoracic or lumbar sympathetic ganglia is limited in its practicability due to its high complication rates and the necessity of experienced surgeons^22^. However, a catheter-based approach may be suboptimal to achieve effective RDN. As discussed earlier, renal sympathetic nerves are abundant around the renal artery in a complex manner. We have reported that renal nerves may not be fully destructed using a catheter-based RDN in humans because of the limited lesion depth of 2 mm^23^. However, 16–30% of renal nerve fibers are located more than 2 mm away from the endoluminal surface of the renal artery^11,24^. To ablate renal nerves located far from the artery, a higher power or longer duration of energy delivery is required for a catheter-based approach. However, increased energy delivery increases the risk of injury to the endoluminal surface and renal artery stenosis. Therefore, a catheter-based RDN may be difficult to completely destroy the renal nerves using catheter-based RDN. Furthermore, a catheter-based approach may not be feasible in cases of severe renal artery stenosis, narrow diameter of the renal artery, significant atherosclerosis or plaques, and anomalous anatomy. Consequently, a novel approach is needed to increase the efficacy and overcome the inherent limitations of a catheter-based approach.

Recently, we developed a novel laparoscopic RDN system^13^. A novel laparoscopy-based approach could be a safer alternative to a surgical approach owing to its minimal invasiveness. Moreover, it can achieve a more effective RDN than a catheter-based approach, considering the distribution of the renal sympathetic nerve. In addition to the surgical dissection of the visible renal nerves, we circumferentially ablated the periarterial nerve fibers using radiofrequency energy. The laparoscopic RDN protocol of maintaining a tip temperature of 65 °C for 70 s to ablate renal nerves has proven effective and safe in our previous studies^13^. Using the laparoscopic system, we have shown that complete RDN could be achieved in the main renal artery and its branches^12^. Accordingly, our analysis also showed that the renal nerve fibers could be effectively destructed without resulting in significant arterial injuries (Fig. 3). Therefore, we believe that the laparoscopic approach could be an attractive alternative to the catheter-based approach.

### Limitations

This study has the following limitations. First, the small sample size may limit the interpretation of the results. Therefore, our study should be interpreted as a proof-of-concept animal study. Second, this study did not measure dispersion in AERP, which may also have affected AF inducibility. Third, all experiments were conducted under general anesthesia; thus, the results may differ in an ambulatory setting. The use of sedatives and analgesics may have affected the experimental results. Fourth, although each swine underwent general anesthesia with a unified protocol and analgesics, the effect of anesthesia might have varied across the subjects. Although the level of anesthesia can influence the autonomic nervous system and affect both inducibility and durations of AF, we could not evaluate the effect of anesthesia using these variables. Fifth, investigation of the long-term impact of laparoscopic RDN on AF inducibility requires further study. Sixth, this study used healthy swine, and the results could be different if there is a cardiovascular or autonomic disorder. Seventh, sympathetic or parasympathetic nerve activity from the stellate ganglion or vagus nerve was not recorded during the experiment. Eighth, although our study did not report any periprocedural complications, we cannot guarantee that laparoscopic RDN is immune to safety concerns. The sample size may be insufficient to affirm the procedure's safety, and long-term follow-up data were unavailable. Further clarification of the detailed mechanism of the acute effect of laparoscopic RDN on AF inducibility is required. Lastly, this study did not perform a head-to-head comparison between catheter-based and laparoscopic approaches for RDN. Therefore, a comparison of the efficacy and safety of the two methods should be performed in future studies.

## Conclusions

This study demonstrated that a novel laparoscopic RDN immediately reduced AF inducibility in a swine model. The acute impact of laparoscopic RDN on increasing AERP and reducing AF inducibility became prominent with increased vagal tone. These results suggest that laparoscopic RDN may decrease the occurrence of AF, especially in the vagally mediated type. In addition, laparoscopic RDN effectively destroyed the renal nerves while preserving the renal artery structure without causing significant vessel injuries. Further studies are warranted to validate the therapeutic effectiveness and safety of AF and its long-term outcomes.

## Methods

Fourteen swine were used in the experiment. Two swine were used to test and establish protocols for vagus nerve stimulation and electrophysiological studies. Three swine were used to test laparoscopic RDN. Finally, nine swine were randomly allocated to the sham or RDN group. All experiments were approved by the Institutional Animal Care and Use Committee of Seoul National University Hospital (SNUH-IACUC), and the animals were maintained in a facility accredited by AAALAC International (no. 001169) in accordance with the Guide for the Care and Use of Laboratory Animals, 8^th^ edition, National Research Council (2010). The study was reported in accordance with the ARRIVE guidelines 2.0.

### Animal preparation

The swine were fasted for 12 h before the experiment. General anesthesia was induced by intramuscular injection of zoletil (5 mg/kg) and xylazine (2 mg/kg). After induction, tracheal intubation was performed using an endotracheal tube and anesthesia was maintained using 2.0–2.5% of isoflurane inhalation during the experiments. Mechanical ventilation was adjusted to maintain oxygen saturation and end-tidal CO_2_ at ≥ 98% and 38–40 mmHg, respectively. Electrodes were attached to the limbs to monitor lead II electrocardiogram (ECG) during the experiment. After anesthesia in the supine position, the neck and abdomen were disinfected with povidone iodine and draped aseptically.

**Figure 4:**
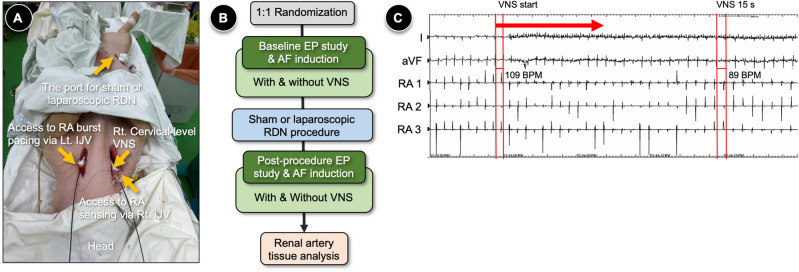
The experimental setup and study flow. (**A**) The experimental setup for EP study and sham or laparoscopic RDN procedure. (**B**) The flowchart of the experiment. (**C**) An example of right cervical VNS. *BPM* beat per minute, *EP* electrophysiology, *IJV* internal jugular vein, *RA* right atrium, *RDN* renal denervation, *VNS* vagal nerve stimulation.

The animal preparation process is illustrated in Fig. 4A. A vertical skin incision was made on both sides of the trachea between the sternohyoid muscle and the medial aspect of the sternocleidomastoid muscle. The subcutaneous tissues were dissected, and the internal jugular vein and the carotid artery were exposed. The right and left jugular veins were punctured using a 7-Fr sheath. A decapolar catheter was introduced into the right atrium via the jugular vein, under fluoroscopic guidance. A decapolar catheter from the right jugular vein was used to record intracardiac electrocardiograms from the lateral side of the right atrium. The other catheter from the left jugular vein was used to perform burst pacing at the right atrium. The pacing output, with a pacing interval of 400 ms, was decreased from 10 to 2 V, and the left decapolar catheter was positioned to maintain stable pacing. Pacing stability was confirmed using both surface ECG and intracardiac electrogram. The right carotid artery was exposed and slightly retracted in order to identify the right vagus nerve.

### Measurement of atrial effective refractory period and AF induction

The experimental flow is presented in Fig. 4B. The capture threshold of the right atrium was tested by decreasing the pacing output from 10 to 1 V. For the remaining experiment, the pacing output was set to be twofold of the capture threshold. After a train of eight S1s at 500 ms, S2 was given from 400 ms and decreased by 10 ms until the AERP was reached. The AERP was measured at the lateral side of the right atrium. The protocol was repeated three times to confirm the AERP. Cardiac pacing and intracardiac electrogram recordings were performed using the Prucka Cardiolab EP System (GE Medical Systems, Fairfield, CT, USA).

AF induction was performed by burst pacing the right atrium at a pacing interval of 100 ms and pulse width of 1.0 ms over 60 s. AF induction was repeated over 10 times with a resting interval of 60 s. Induced AF episodes were confirmed by recording surface ECG and intracardiac electrogram, and the duration of each AF episode was measured. AF episodes were defined as cases lasting ≥ 5 s because supraventricular tachycardia episodes less than 5 s after burst pacing could be non-specific findings. The inducibility of AF was defined as the success rate of the AF induction tests and measured as a percentage.

For both sham and RDN groups, each swine underwent AERP and AF induction tests before and after the procedure (i.e., a sham or laparoscopic RDN procedure for the sham or RDN group, respectively). In addition, the AERP and AF induction tests were repeated with and without VNS.

### Vagal nerve stimulation

Considering that AF may not be readily inducible by right atrial burst pacing for healthy swine, we used the VNS to promote AF inducibility in healthy swine. In this study, both AERP and durations of AF episodes were measured with and without VNS for each swine. The protocol of VNS was as follows. The right cervical vagus nerve was located beneath the carotid artery. A pacing electrode (Model 6491, Unipolar Pediatric Temporary Pacing Lead; Medtronic, Minneapolis, MN, USA) was placed and fixed to the vagus nerve. VNS was performed using a Grass S88 stimulator (A-M Systems). The vagus nerve was stimulated with a pulse width of 0.2 ms and a frequency of 20 Hz. The pacing output for VNS was adjusted to 2–10 V to achieve a 10% decrease in heart rate without significant hemodynamic instability. An example of VNS is illustrated in Fig. 4C. The VNS duration was limited to 30 s to avoid saturation effects.

### Laparoscopic RDN and sham procedures

**Figure 5:**
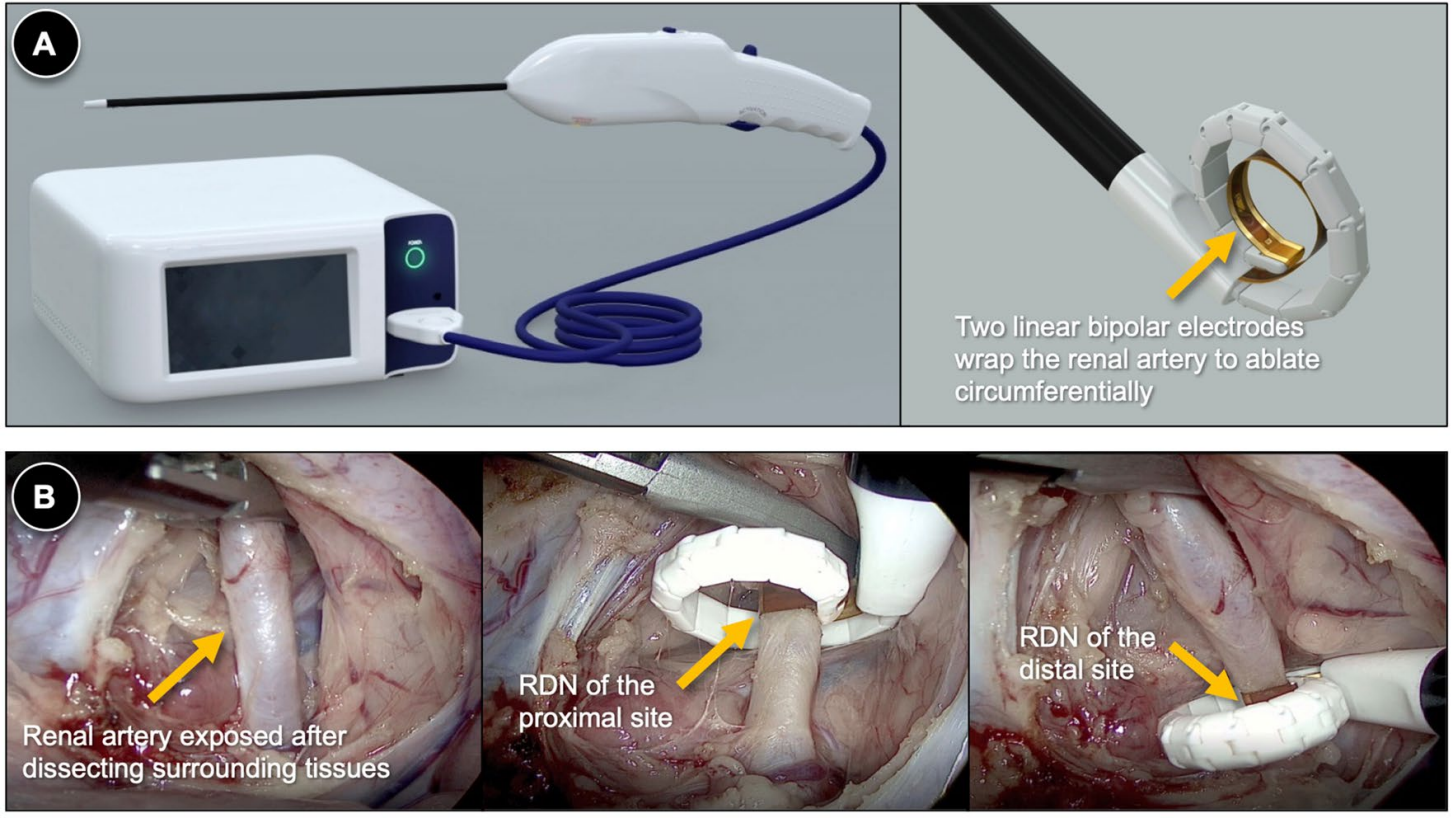
Demonstration of the laparoscopic RDN procedure. (**A**) Schematic illustration of the novel laparoscopic RDN system. The instrument’s tip wraps around the renal artery and delivers radiofrequency energy using a pair of linear bipolar electrodes apart 2 mm away from each other (adapted from *Investig Clin Urol*. 2020;61(1):107–113, the courtesy of Jeong CW, MD, PhD). (**B**) Laparoscopic view of the laparoscopic RDN procedure. The renal artery is exposed after cutting peritoneum and dissecting periarterial subcutaneous tissues (the left panel). After exposing the renal artery, RDN is performed at both the proximal and distal sites of the artery (the middle and right panels). *RDN* renal denervation.

Laparoscopic RDN was performed as follows. After draping, a Veress needle was inserted 5 cm to the left or right and 2 cm caudal to the umbilicus, and CO_2_ was insufflated into the abdominal cavity. Trocars were placed as follows: (1) a 12-mm port 2 cm caudal to the umbilicus and the lateral margin of the rectus muscles (for the camera); (2) a 12-mm port 7 cm cephalad to the camera port; and (3) 5-mm ports 7–8 cm lateral to the camera port. The laparoscopic camera visualized the intra-abdominal cavity. The renal artery was exposed after perirenal fascia incision and dissection of the surrounding soft tissue. Circumferential RDNs were performed using a HyperQure Renal Denervation Laparoscopic Instrument (DeepQure, Inc., Seoul, Republic of Korea) at both the proximal and distal sites of the renal artery with a minimum distance of 3 mm. After wrapping the renal artery using an electrode at the tip of the instrument, bipolar radiofrequency energy was delivered at a constant temperature of 65°C for 70 s using a single shot (Fig. 5). When the distal renal artery bifurcated, each branch was ablated separately. This procedure was repeated for the contralateral kidney. After RDN, the perirenal fascia was sutured, and intraperitoneal CO_2_ was exsufflated. The abdominal walls were closed, and an occlusive dressing was applied. For the sham procedure, all procedures were performed as in the RDN group, except for the delivery of radiofrequency energy.

### Tissue analysis

After the experiment, the swine were fully sedated with general anesthesia using 4–5% of isoflurane inhalation and euthanized by an intravenous potassium chloride (2 mmol/kg) injection through the internal jugular vein. Subsequently, both left and right renal arteries were collected. The renal artery was fixed with 10% formalin and stained with hematoxylin and eosin. After paraffin embedding, 4 μm-thickness horizontal sections of the renal artery were obtained at both proximal and distal ablated sites. Immunohistochemistry was performed using a mouse monoclonal antibody against TH (Accurate Chemical & Scientific Corporation, Carle Place, NY, USA) to visualize the renal sympathetic nerves. Nerve fibers were counted along the adventitia of the renal artery and compared between the sham and RDN groups.

### Statistical analysis

According to variable types, data are shown as n (%), mean ± standard deviation, or median (interquartile range). For each swine, the Wilcoxon signed-rank test was performed to compare the pair of AERPs (or AF durations) obtained before and after the sham or laparoscopic RDN procedure. The association between AF inducibility and procedures (sham or laparoscopic RDN) was evaluated by calculating odds ratios (ORs) with 95% confidence intervals (CIs). The Mann–Whitney *U*-test was performed to compare nerve fiber counts between the sham and RDN groups. Two-sided p-values less than 0.05 assumed the rejection of the null hypothesis. All statistical analyses were performed using the IBM SPSS Statistics for Windows (version 22.0; IBM Corp., Armonk, NY, USA).

### Supplementary Information

Supplementary Figure 1.

## Data Availability

The data underlying this article will be shared on reasonable request to the corresponding author.

## Abbreviations

AERP: Atrial effective refractory period
AF: Atrial fibrillation
RDN: Renal denervation
VNS: Vagal nerve stimulation
TH: Tyrosine hydroxylase
