# Supplementary material for: Novel laparoscopic renal denervation immediately reduces atrial fibrillation inducibility: a swine model study
Source: Sci Rep. 2023 Nov 11;13:19679. doi: 10.1038/s41598-023-47077-w (PMC10640613; doi:10.1038/s41598-023-47077-w)

**Supplemental Appendix**

**Novel Laparoscopic Renal Denervation Immediately Reduces Atrial Fibrillation Inducibility: A Swine Model Study**

Soonil Kwon, MD,^a^ Eue-Keun Choi, MD, PhD,^a,b,*^ Hyo-Jeong Ahn, MD,^a^ So-Ryoung Lee, MD, PhD,^a^ and Seil Oh, MD, PhD,^a,b^ Si Hyun Kim, MD,^c^ Minh-Tung Do, MD,^c,d^ Jang Hee Han, MD, PhD,^c^ Chang Wook Jeong, MD, PhD^c,e,*^

^a^ Division of Cardiology, Department of Internal Medicine, Seoul National University Hospital, Seoul, Republic of Korea

^b^ Department of Internal Medicine, Seoul National University College of Medicine, Seoul, Republic of Korea

^c^ Department of Urology, Seoul National University Hospital, Seoul, Republic of Korea

^d^ Department of Surgery, Hai Phong University of Medicine and Pharmacy, Hai Phong, Vietnam

^e^ Department of Urology, Seoul National University College of Medicine, Seoul, Republic of Korea

^*^ Co-corresponding authors.

Supplemental Figure 1. Representative cross-sectional slides of (A) proximal and (B) distal sites of the renal artery.


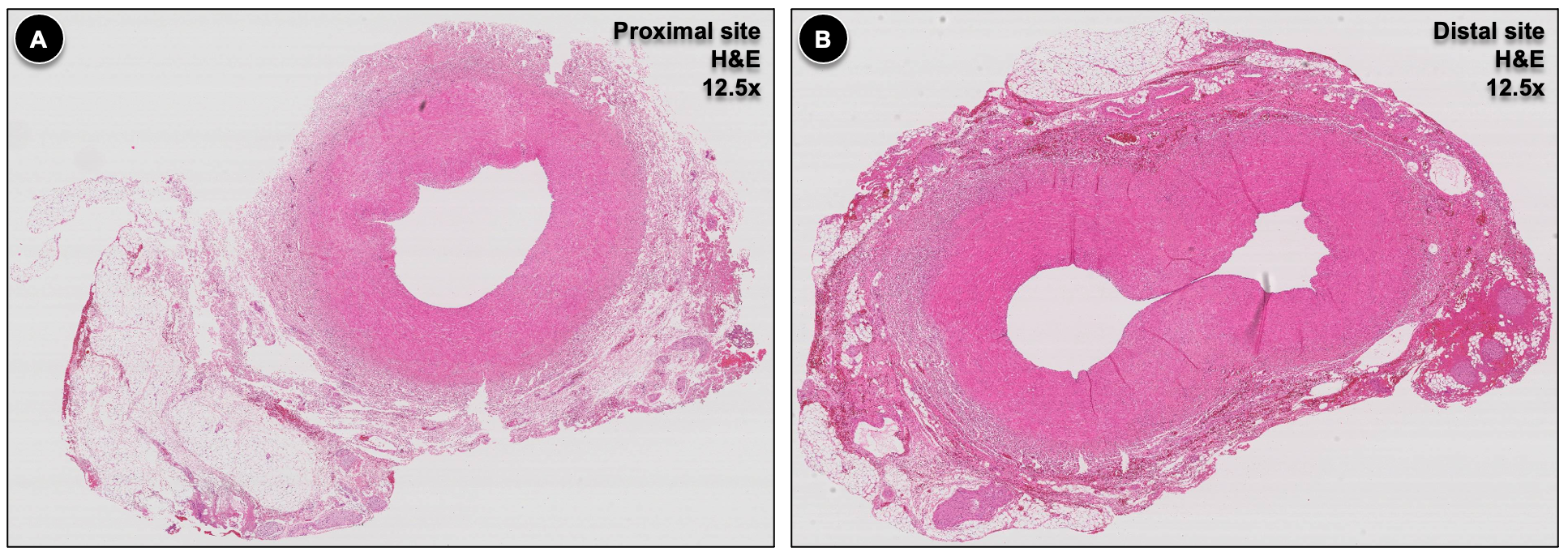

Supplement: Supplementary file 1 — Supplementary Figure 1. [file 41598_2023_47077_MOESM1_ESM.docx]
